# Deep Sequencing Revealed a CpG Methylation Pattern Associated With *ALDH1L1* Suppression in Breast Cancer

**DOI:** 10.3389/fgene.2018.00169

**Published:** 2018-05-15

**Authors:** Artemy D. Beniaminov, Grigory A. Puzanov, George S. Krasnov, Dmitry N. Kaluzhny, Tatiana P. Kazubskaya, Eleonora A. Braga, Anna V. Kudryavtseva, Nataliya V. Melnikova, Alexey A. Dmitriev

**Affiliations:** ^1^Engelhardt Institute of Molecular Biology, Russian Academy of Sciences, Moscow, Russia; ^2^N.N. Blokhin National Medical Research Center of Oncology, Moscow, Russia; ^3^Institute of General Pathology and Pathophysiology, Moscow, Russia

**Keywords:** *ALDH1L1*, breast cancer, expression downregulation, DNA methylation, deep sequencing, qPCR

## Abstract

Hypermethylation of promoter CpG islands is generally recognized epigenetic mechanism responsible for gene silencing in cancer. However, molecular details on how this epigenetic mark triggers the process of gene downregulation are still elusive. Here, we used deep bisulfite sequencing and qPCR analysis to investigate the pattern of CpG methylation of *ALDH1L1* promoter region and its association with the gene expression level in 16 paired breast cancer (BC) samples of different clinical stages. Expression of *ALDH1L1* gene was suppressed in all examined BC samples up to 200-fold, and average hypermethylation level of the promoter region correlated positively with *ALDH1L1* downregulation. We determined the role of every individual CpG site within the *ALDH1L1* promoter, including upstream untranscribed region, first untranslated exon, and the start of the first intron, in aberrant gene expression by correlation analysis. The search revealed CpG sites which methylation has the highest impact on intensity of gene transcription. The majority of such CpG sites are located in a compact region in the first intron of the *ALDH1L1* gene. These results assist in unraveling of dynamic nature of CpG promoter hypermethylation as well as demonstrate the efficiency of deep bisulfite sequencing in search for novel epigenetic markers in cancer.

## Introduction

Significantly altered protein expression pattern distinguishes cancer cells from normal ones. These changes concern not only tumor suppressors or oncogene proteins but a variety of cellular proteins and believed to provide a selective advantage for uncontrolled proliferation, one of the hallmarks of cancer (Hanahan and Weinberg, 2000; Hanash and Taguchi, 2010). Expression of a particular gene can exhibit both up- and down-regulation depending on a cancer type, histological subtype, stage of tumor development. However, certain proteins, like tumor suppressors, are downregulated in the majority of tumors. Thereby, inactivation or suppression of corresponding genes presumably remove negative regulation of cell growth and contribute to the abnormal proliferation of tumor cells.

*ALDH1L1* (aldehyde dehydrogenase 1 family, member L1) is one of the genes which expression is strongly downregulated in many human cancers including hepatocellular carcinoma, pilocytic astrocytoma, liver cancer (Krupenko and Oleinik, 2002; Rodriguez et al., 2008; Chen et al., 2012), renal cell carcinoma (Dmitriev et al., 2014), lung adenocarcinoma (Oleinik et al., 2011). The product of the *ALDH1L1* gene, 10-formyltetrahydrofolate dehydrogenase (FDH), is an abundant cytosolic enzyme involved in folate pathways (Krupenko, 2009). The enzyme belongs to the aldehyde dehydrogenase family and catalyzes the conversion of 10-formyltetrahydrofolate, NADP, and water to tetrahydrofolate, NADPH, and carbon dioxide. Abundance of FDH in several normal tissues (Kutzbach and Stokstad, 1971) implies the importance of folate pathway for cellular functions. The observation that FDH is a strong marker of astrocytes in the rat brain suggests a function for the enzyme in the nervous system (Cahoy et al., 2008).

Tissue-specific expression of *ALDH1L1* and its strong suppression in certain cancer types suggest that the gene is tightly regulated (Krupenko and Oleinik, 2002). Theoretically, different mechanisms can control expression of *ALDH1L1* in the cell including epigenetic silencing, transcription factor- or microRNA-mediated suppression, and mutations. One indication that epigenetic mechanisms and specifically CpG methylation could be involved in control of *ALDH1L1* expression emerged from chromosome 3 studies by NotI-microarrays in non-small cell lung cancer (Dmitriev et al., 2012), cervical cancer (Senchenko et al., 2013), and clear cell renal cell carcinoma (Dmitriev et al., 2014). Other works demonstrated the importance of CpG methylation in suppression of *ALDH1L1* in several cancer types and cell lines including lung adenocarcinoma, hepatocellular carcinoma (Oleinik et al., 2011), and esophageal squamous cell carcinoma (Chen et al., 2015). Cancer cell lines A549, HepG2, and HCT116 revealed high degree of *ALDH1L1* promoter methylation, and treatment of FDH-deficient A549 cells with the methyltransferase inhibitor 5-aza-2′-deoxycytidine restored FDH expression (Oleinik et al., 2011).

On the whole, these data indicate that promoter hypermethylation might be a common mechanism of *ALDH1L1* downregulation in human cancers. Methylation is a well-studied mechanism of gene silencing in cancer, and a considerable list of genes which suppression is associated with hypermethylation is known to date. However, the details of this regulatory process have yet to be elucidated. Those include the dynamics of promoter methylation with tumor progression, resolution of specific CpG sites responsible for silencing of different genes in different cancer types, *etc*. To elaborate on the detailed pattern of promoter methylation in cancer, representative samplings of tumor DNA should be considered and modern deep bisulfite sequencing techniques should be applied. In this work, we demonstrate that *ALDH1L1* gene is strongly downregulated in breast cancer (BC) and interrogate the role of methylation status of each individual CpG site within the promoter region in suppression of the gene. To this end, we have performed in-depth analysis of methylation pattern in the region encompassing upstream untranscribed region, the first exon, and the first intron of *ALDH1L1* gene.

## Materials and Methods

### Human Tissue Samples

Sixteen paired (tumor/normal) specimens were obtained after surgical resection from patients diagnosed with BC prior to radiation or chemotherapy and stored in liquid nitrogen. The diagnosis was verified by histopathology and only samples containing at least 70–80% tumor cells were used in the study. The samples were collected in accordance with the guidelines issued by the Ethics Committee of N.N. Blokhin National Medical Research Center of Oncology (Moscow, Russia). The Ethics Committee of N.N. Blokhin National Medical Research Center of Oncology specifically approved this study. All patients gave written informed consent in accordance with the principles outlined in the Declaration of Helsinki.

### Genomic DNA and Total RNA Isolation

Prior to DNA or RNA isolation, the tissue samples were ground and homogenized in liquid nitrogen. Genomic DNA was extracted and purified using QIAamp DNA Mini Kit (Qiagen, United States) according to manufacturer’s guidelines. Control samples of Jurkat Genomic DNA and CpG Methylated Jurkat Genomic DNA were purchased from Thermo Fisher Scientific (United States).

Total RNA was isolated from 10 to 30 mg of tissue samples using RNeasy Mini Kit (Qiagen). The quality of the RNA samples was monitored by absorption ratio at A260/A280 and electrophoretically by the ratio of band intensities for 28S *vs.* 18S rRNA in agarose gel or using Agilent 2100 Bioanalyzer (Agilent Technologies, United States).

### Reverse Transcription and qPCR

For reverse transcription, 1 μg of total RNA was converted to cDNA using random hexamer primers and RevertAid reverse transcriptase (Thermo Fisher Scientific) according to the manufacturer’s protocol. The mRNA level of the target gene *ALDH1L1* was measured by the TaqMan technique using 7500 Real-Time PCR System (Applied Biosystems, United States) in a total volume of 25 μl. Thermo Fisher Scientific commercial primer-probe sets were applied to evaluate expression level of *ALDH1L1* gene (Hs01003842_m1) as well as *B2M* and *RPN1* genes (Hs00187842_m1 and Hs00161446_m1, respectively). The latter served as reference genes for normalization of the quantitative data. RT-minus controls were used for every sample and each pair of primers to exclude possible contamination. All measurements were done in triplicate. Quantitative PCR (qPCR) data were analyzed using the relative quantification method, or ΔΔCt-method (Schmittgen and Livak, 2008), that is based on the comparison of target and reference genes in tumor (T) and normal tissue (N) samples. All calculations were performed using our ATG tool (Melnikova et al., 2016) as described earlier (Dmitriev et al., 2016).

### Bisulfite Conversion and Sequencing

Bisulfite-mediated conversion of unmethylated cytosines to uracils of the extracted or commercially available genomic DNA (1 μg) was performed with EZ DNA Methylation-Gold Kit (Zymo Research, United States) according to the manufacturer’s directions. After conversion, three overlapping fragments of the promoter region of *ALDH1L1* gene were amplified by PCR using specific primers designed by Oleinik et al. (2011). These locus-specific primers were tagged at 5′-end with universal Illumina adapter overhang sequences: TCGTCGGCAGCGTCAGATGTGTATAAGAGACAG for forward primers and GTCTCGTGGGCTCGGAGATGTGTATAAGAGACAG for reverse primers to obtain three pairs of oligos (Supplementary Table 1). Three amplified DNA fragments were combined, cleaned up using Agencourt AMPure XP PCR purification system (Beckman Coulter, United States), followed by Index PCR amplification using Nextera XT Index primers (Supplementary Table 1). After the second round of purification, DNA concentration and quality of the final library were assessed using Agilent 2100 Bioanalyzer.

Sequencing was performed using MiSeq (Illumina). For each sample, more than 100,000 reads were obtained. Trimming, reference alignment, and C to T conversion analysis were performed using CLC Genomics WorkBench (Qiagen). Efficiency and specificity of bisulfite conversion of the genomic DNA were monitored as follows. Conversion of non-CpG cytosines to thymines exceeded 99% in all the samples evaluated. At the same time, 97% of CpG sites in the analyzed genomic fragment of CpG Methylated Jurkat Genomic DNA remained unconverted to thymines after bisulfite treatment.

Methylation level for each individual CpG site was defined as a ratio of number of reads contained non-converted cytosine in the CpG site to total number of reads covering this CpG site. For *ΔM* calculation, methylation level of a CpG site in a normal tissue sample was subtracted from that in matched tumor sample.

### Bioinformatics Analysis of TCGA Data

The Cancer Genome Atlas (TCGA^1^) database analysis was performed to identify changes in expression and methylation profiles of *ALDH1L1* gene in BC. The search through TCGA identified the data on DNA methylation (Illumina Infinium HumanMethylation450K BeadChip platform) and transcriptome sequencing (RNA-Seq, Illumina HiSeq 2000/2500 systems) for 786 tumor and 84 histologically normal breast samples (The Cancer Genome Atlas Network, 2012; Ciriello et al., 2015). These data and CrossHub application (Krasnov et al., 2016) were used to evaluate methylation patterns and expression profile of *ALDH1L1* and to search for correlations between expression and methylation of CpG sites of the gene.

This work was performed using the equipment of “Genome” center of Engelhardt Institute of Molecular Biology^2^.

## Results

### Expression Profile of *ALDH1L1* Gene in Breast Cancer

**FIGURE 1:**
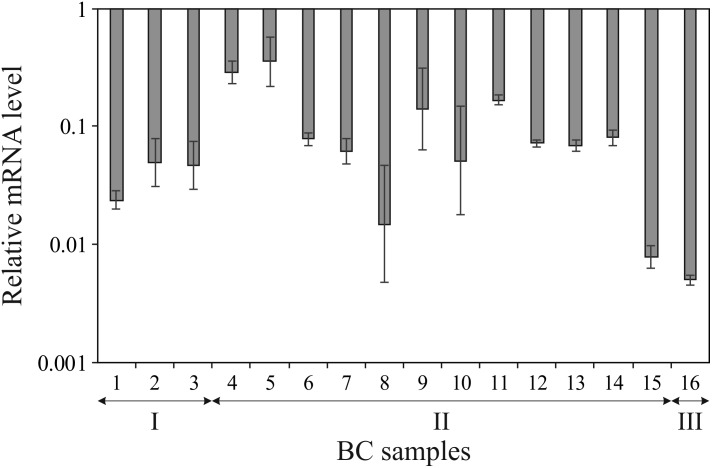
Downregulation of *ALDH1L1* gene in breast cancer (BC). Decrease in *ALDH1L1* mRNA level is shown in logarithmic scale for each tumor sample compared to the matched normal tissue sample as derived from qPCR data. Experiments were performed in triplicate and error bars show standard deviations. BC samples are grouped according to clinical stages (I, II, and III).

We have determined expression of *ALDH1L1* gene in 16 paired ÂÑ samples using qPCR. Drastic downregulation was revealed in all tumor specimens compared to their normal counterparts: the mRNA level decrease ranged from 3- to 200-fold with median equaled 15 (**Figure 1**). Statistical significance of the difference in *ALDH1L1* level between tumor and normal tissues for 16 paired samples was assessed by the Mann–Whitney test: *p* < 0.02. No apparent correlation was observed between gene expression and clinical stage, although the sampling was relatively small and distribution of samples between the stages was uneven to address such a question.

### Analysis of Data From TCGA and ENCODE Projects

**FIGURE 2:**
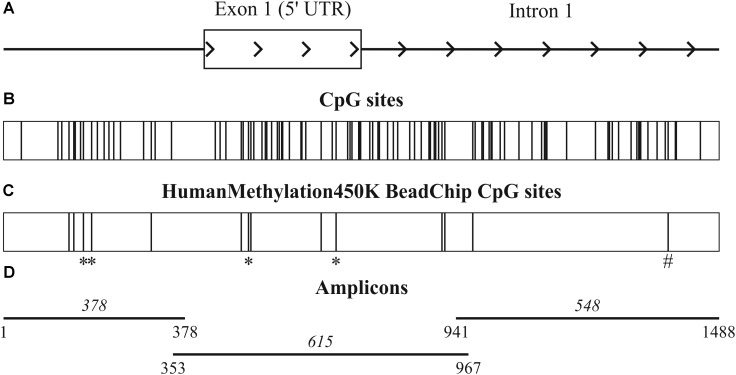
Promoter region of *ALDH1L1* gene. **(A)** 1488 bp promoter region of *ALDH1L1* gene (hg19, chr3:125,898,575–125,900,062) includes untranscribed region, the first exon, and the beginning of the first intron. **(B)** Total 97 CpG sites of the region include **(C)** 14 CpG sites covered by Infinium HumanMethylation450K BeadChip. Four CpG sites with high hypermethylation score are marked with an asterisks and one CpG site, which hypermethylation correlates with suppression of *ALDH1L1*, is marked with a hash symbol. **(D)** Three overlapping amplicons used for bisulfite sequencing and their sizes in base pairs are shown.

In order to define possible causes for such downregulation of *ALDH1L1* in BC, we applied CrossHub tool recently developed by us (Krasnov et al., 2016). CrossHub predicts the contribution of various genetic and epigenetic mechanisms [including transcription factors (TFs), microRNA, and CpG methylation] to control of gene expression by collecting and analyzing the data available from TCGA, Encyclopedia of DNA Elements (ENCODE), and Gene Ontology (GO) projects, as well as from microRNA-mRNA and TF-DNA predictive and experiment-based databases. The bioinformatics analysis pointed to the dominating role of CpG methylation in regulation of *ALDH1L1* expression in BC. The promoter region of *ALDH1L1* spans nucleotide positions 125,898,681–125,899,926 of chromosome 3 (**Figure 2A**) in the human genome (hg19) and is marked as TSS (predicted promoter region including TSS) for two cell lines, H1-hESC and HepG2, as derived from combined segmentation track of the ENCODE project. The region includes 14 CpG sites that are covered by Infinium HumanMethylation450K BeadChip being distributed between upstream untranscribed region (5 CpG), first untranslated exon (5 CpG), and the beginning of the first intron (4 CpG) as depicted in **Figures 2B,C**.

Being essentially unmethylated in normal tissue samples, the 14 CpG sites exhibited different extent of methylation in tumors. Methylation status of four CpG sites (cg01566526, cg07034362, cg11022432, cg16771578, Infinium HumanMethylation450K BeadChip data numbering) demonstrated the highest hypermethylation score (according to CrossHub calculation), which reflected the frequency and extent of hypermethylation in BC samples (**Figure 2C**, marked with an asterisk). At the same time, high hypermethylation score of a CpG site was not necessarily resulted in strong negative correlation between hypermethylation of this site in cancer and relative expression level of *ALDH1L1* gene. On the contrary, the only CpG site (cg27282530) that demonstrated statistically significant correlation of this type (*p* = 0.02) belonged to the first intron of *ALDH1L1* gene (**Figure 2C**, marked with a hash symbol).

In summary, these results strongly argued for the prevailing role of CpG hypermethylation of *ALDH1L1* promoter region in suppression of the gene in BC. Although the connection between methylation of promoter and expression of *ALDH1L1* appears very plausible, it is not obvious whether methylation status of certain individual CpG sites or average methylation level of the entire gene promoter is responsible for the decrease of the mRNA level. To address this question, we performed deep bisulfite sequencing of *ALDH1L1* promoter region.

### Pattern of Hypermethylation of *ALDH1L1* Promoter Region in Breast Cancer

**FIGURE 3:**
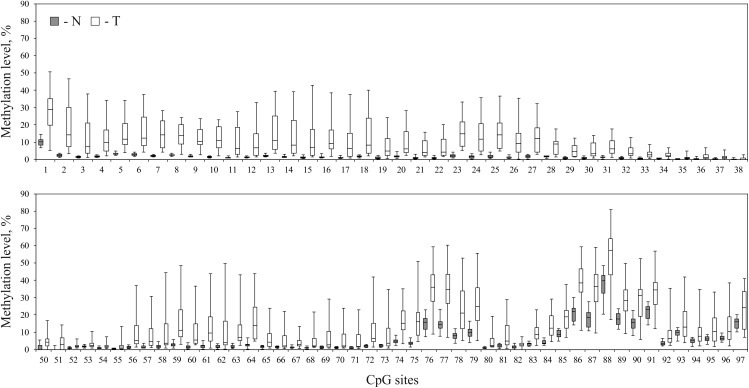
Comparison of methylation patterns in normal and tumor breast tissue samples. Methylation level of every CpG site within *ALDH1L1* promoter region of 1488 nucleotides (hg19, chr3:125,898,575–125,900,062) in normal (N, filled rectangles) and tumor (T, open rectangles) tissues of patients with BC. CpG sites 39–49 were omitted from the analysis due to low coverage. Rectangles correspond to the ranges containing 50% of the values (between the 25th and 75th percentiles); the horizontal line inside the rectangle is the median value (the 50th percentile); the bars are the maximum and minimum values.

**FIGURE 4:**
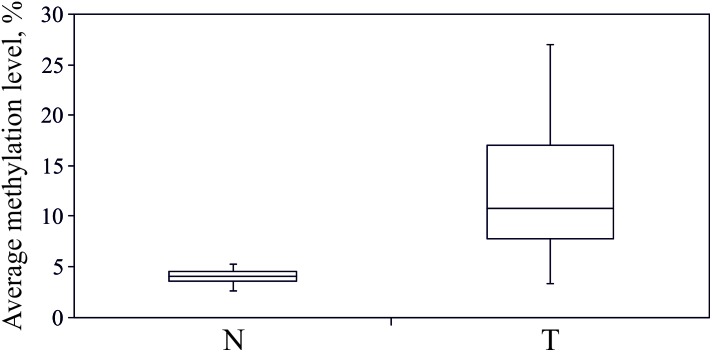
Hypermethylation of *ALDH1L1* promoter region in BC. Average (for 97 CpG sites) methylation level of *ALDH1L1* promoter region in normal (N) and tumor (T) tissues of patients with BC. Deep sequencing data. Rectangles correspond to the ranges containing 50% of the values (between the 25th and 75th percentiles); the horizontal line inside the rectangle is the median value (the 50th percentile); the bars are the maximum and minimum values.

As revealed from the analysis of TCGA project data (Infinium HumanMethylation450K BeadChip data, BC samples), hypermethylation should affect upstream untranscribed region, the first exon of the gene, which is entirely untranslated, and the start of the first intron. We performed bisulfite conversion of genomic DNA and amplified three overlapping fragments by PCR to cover the entire genomic region. The three amplicons covered on the whole 1488 nucleotides (hg19, chr3:125,898,575–125,900,062) and included 97 CpG sites (**Figure 2D**). Sequencing was performed on MiSeq platform which generated paired-end reads of 300 bp. The 1488-bp-long fragment was covered by at least 100,000 reads for each of the 16 paired BC samples (the raw data were deposited in Sequence Read Archive – SRP142591). The size of the second amplicon exceeded 600 bp, thereby the center of the amplicon was poorly covered. The CpG sites (numbers 39–49) were covered by less than 10 trimmed reads in at least one tumor or normal tissue sample and therefore were excluded from the analysis. Non-CpG promoter methylation was recently reported to be a key factor in downregulation of some tumor suppressor genes, e.g., O6-methylguanine-DNA methyltransferase gene and runt-related transcription factor 3 (Saikia et al., 2017). This type of modification is unlikely involved in regulation of *ALDH1L1* gene in BC since we failed to determine any hypermethylation of non-CpG sites. All non-CpG cytosines were detected as thymines in more than 99% of reads both in tumor and normal tissue samples, and therefore they were essentially unmethylated, that also pointed to high efficiency of bisulfite conversion. Methylation level was calculated (see section “Materials and Methods”) for each individual CpG site within *ALDH1L1* promoter region (**Figure 3**). Normal tissue samples demonstrated low methylation level (4% on average, **Figure 4**) that can indicate basic methylation level of CpG island in genomic DNA of normal tissue or contamination of the normal tissue samples with tumor cells. Methylation level of the region in tumor samples was significantly greater (11% on average, *p* < 0.02, Mann–Whitney test, **Figure 4**) and revealed high degree of heterogeneity both between the samples and positions of CpG sites (**Figure 3**). Five BC samples showed no significant increase in methylation level: average *ΔM* for all CpG sites in a sample was less than 5%. Since mRNA level of *ALDH1L1* was still sizably decreased in these BC samples, other downregulation mechanisms should be considered. The remaining 11 BC samples were hypermethylated by up to 25% (on average for all CpG sites in a sample).

**FIGURE 5:**
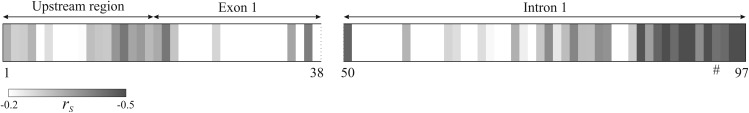
Correlation between CpG promoter hypermethylation and relative mRNA level of *ALDH1L1* gene in BC. For each individual CpG site (1–97), Spearman’s correlation coefficient (*r_s_*) between *ΔM* and *ALDH1L1* relative expression level is represented by a shade of gray: from white (*r_s_* ≥ –0.2) to dark gray (*r_s_* ≤ –0.5). No positive *r_s_* values greater than 0.15 were observed. The gap for CpG sites 39–49 corresponds to unreliable or unavailable methylation data (see explanations in the text). Hash symbol marks CpG site #94 which methylation negatively correlated with *ALDH1L1* expression according to Infinium HumanMethylation450K BeadChip data analysis for BC.

We compared how relative expression level of *ALDH1L1* correlates with average hypermethylation of the analyzed promoter region and with hypermethylation of every individual CpG site of this genomic fragment in BC. The average methylation level for all CpG sites in a sample revealed negative correlation with relative mRNA level of *ALDH1L1* gene in the examined sampling: Spearman’s correlation coefficient (*r_s_*) was equal to -0.38 (*p* = 0.14). Correlation between the relative gene expression and hypermethylation of individual CpG sites was predominantly negative with *r_s_* down to -0.57 (*p* = 0.02, **Figure 5**). No CpG sites with significant positive correlation were detected: positive *r_s_* values for a few CpG sites were observed but turned out to be less than 0.15. Apparently, CpG sites 85–97, which belong to the first intron of the gene, reflect suppression of *ALDH1L1* most reliably as having the highest correlation coefficients within the promoter region of the gene. The only CpG site (cg27282530) identified by us previously in Infinium HumanMethylation450K data for BC as having considerable negative correlation with *ALDH1L1* gene expression (*r_s_* = -0.27, *p* = 0.02) also belongs to this peak region (CpG #94, marked with a hash symbol, **Figure 5**). At the same time, CpG sites 86–91 had the highest level of hypermethylation in BC samples and thereby are the best candidates for epigenetic markers of tumor cells (**Figure 3**).

## Discussion

In this work, we have demonstrated that *ALDH1L1* gene was strongly suppressed in 100% of BC samples. Similar drop in *ALDH1L1* expression had been observed in the majority of other malignant tumors tested so far (Krupenko and Oleinik, 2002; Rodriguez et al., 2008; Oleinik et al., 2011; Chen et al., 2012; Dmitriev et al., 2014). Our analysis of TCGA data showed that suppression of the gene in BC is accompanied by CpG hypermethylation of its promoter region. Accordingly, several studies had previously reported about aberrant methylation of *ALDH1L1* promoter in lung adenocarcinoma, hepatocellular carcinoma (Oleinik et al., 2011), and esophageal squamous cell carcinoma (Chen et al., 2015), supporting the idea that hypermethylation of *ALDH1L1* is a major mechanism for suppression of the gene in cancer.

The role of promoter hypermethylation in cancer is being intensively studied. More than 60% of all genes have CpG islands and certain fraction (up to 10%) of them can be hypermethylated in cancer (Baylin and Jones, 2016). Some of them may function as tumor suppressor genes, but discerning those which contribute directly to tumorigenesis is a difficult task. Currently, it cannot be said with certainty whether *ALDH1L1* is a *bona fide* tumor suppressor gene or a mere “passenger” which promoter is frequently hypermethylated in cancer. However, *ALDH1L1* suppression in 100% of BC samples allowed us to address several questions on the dynamic and pattern of promoter methylation process. Do *ALDH1L1* promoter methylation level or gene expression level correlate with clinical stage of breast cancer? Which CpG sites are essential for *ALDH1L1* suppression? Is hypermethylation of specific CpG sites or average promoter methylation level responsible for the gene suppression? Answering these questions regarding even one particular gene requires (i) representative sampling of paired cancer specimens and (ii) a high accuracy method for measuring the methylation level in the region of interest.

Regarding temporal aspect, we have not observed any correlation between *ALDH1L1* expression level or its promoter methylation level and BC stage. On one hand, hypermethylation and silencing of the gene even at early stages can indicate that silencing of *ALDH1L1* is prerequisite for malignant transformation and the gene can be considered a tumor suppressor. On the other hand, the result may stem from the sampling size and may change when larger set of paired BC specimens is examined.

The number of methylated CpG sites required for silencing of a gene and their location can differ depending on a specific gene (Tirado-Magallanes et al., 2017). Oleinik et al. (2011) applied bisulfite conversion of DNA from 10 lung adenocarcinoma samples and conventional sequencing of upstream region, the first exon and the beginning of the first intron of *ALDH1L1*. Certain hypermethylation was observed in all three regions with no considerable preference. Although, the importance of the first exon of *ALDH1L1* was emphasized in the experiments, since the exon was most extensively methylated in samples with a strongly downregulated *ALDH1L1* gene, and also the addition of exon 1 to the reporter vector considerably enhanced luciferase expression.

In the current study, we examined the same genomic region containing 97 CpG sites and applied deep sequencing of bisulfite-converted DNA. Bisulfite sequencing is the gold-standard method for detection of CpG methylation in genomic DNA. Contrary to microarray-based techniques, sequencing produces methylation data with individual CpG resolution and does not suffer from possible errors introduced by probe cross-hybridization (Chatterjee et al., 2017). Pyrosequencing and conventional analysis of colonies are two most commonly used sequencing approaches of bisulfite-converted DNA. However, application of deep sequencing to bisulfite-converted DNA provides unprecedented accuracy in measurement of methylation level at every CpG site and allows monitoring of tiny differences between tumor and normal tissues. Due to the high read coverage of the examined 1.5-kb promoter region, we were able to interrogate each CpG site by correlation analysis and identify those CpG sites which methylation had the highest impact on *ALDH1L1* expression. A compact region at the beginning of the first intron of *ALDH1L1* gene revealed the highest negative correlation with gene expression. Second, much less profound peak belonged to untranscribed promoter region and located close to transcription start site. CpG sites of the exon 1 were not revealed in this analysis. The discrepancy of this result with the observation of Oleinik et al. (2011) can be attributed to different tumor type (breast cancer *versus* lung adenocarcinoma) or experimental technique (deep sequencing *versus* conventional sequencing of several clones).

Overall, in this study, we have demonstrated that strong suppression of *ALDH1L1* gene in BC correlated with its CpG island hypermethylation. We were able to discern the first intron as the region most affected by hypermethylation. However, methylation appeared quite heterogeneous between BC samples: several tumor samples revealed no increase in methylation of CpG island. This circumstance argues that hypermethylation is not the only mechanism responsible for *ALDH1L1* downregulation in BC.

## Author Contributions

AB, AK, NM, and AD conceived and designed the research. TK and EB collected and characterized tissue samples. AB, GP, and NM performed the experiments. AB, GK, DK, and AD processed the data, performed statistical analysis and bioinformatics search. AB, NM, and AD wrote the paper. All authors agreed with the final version of the manuscript and all aspects of the work.

## Conflict of Interest Statement

The authors declare that the research was conducted in the absence of any commercial or financial relationships that could be construed as a potential conflict of interest.

## Footnotes

1 https://cancergenome.nih.gov/

2 http://www.eimb.ru/rus/ckp/ccu_genome_c.php
