# Supplementary material for: Deep Sequencing Revealed a CpG Methylation Pattern Associated With ALDH1L1 Suppression in Breast Cancer
Source: Front Genet. 2018 May 15;9:169. doi: 10.3389/fgene.2018.00169 (PMC5962711; doi:10.3389/fgene.2018.00169)
Supplement: Supplementary file 1 [file Table_1.DOCX]

Supplementary Material

**Deep sequencing revealed a CpG methylation pattern associated with *ALDH1L1* suppression in breast cancer**

**Artemy D. Beniaminov*, Grigory A. Puzanov, George S. Krasnov, Dmitry N. Kaluzhny, Tatiana P. Kazubskaya, Eleonora A. Braga, Anna V. Kudryavtseva, Nataliya V. Melnikova, Alexey A. Dmitriev**

*** Correspondence:** Artemy D. Beniaminov: abeniaminov@mail.ru

**Supplementary Table 1. Primers for DNA library preparation.**

| **Primer name** | **Primer sequence** |
| --- | --- |
| ALD_1F | **TCGTCGGCAGCGTCAGATGTGTATAAGAGACAG***TTGGTTAGGATTTTTGGGAATTAGG* |
| ALD_1R | **GTCTCGTGGGCTCGGAGATGTGTATAAGAGACAG***ACACCCAACAAAACTAACAATTCTA* |
| ALD_2F | **TCGTCGGCAGCGTCAGATGTGTATAAGAGACAG***TAGAATTGTTAGTTTTGTTGGGTGT* |
| ALD_2R | **GTCTCGTGGGCTCGGAGATGTGTATAAGAGACAG***ACCTAATAACCCCTAACCAAATTCAA* |
| ALD_3F | **TCGTCGGCAGCGTCAGATGTGTATAAGAGACAG***TTGAATTTGGTTAGGGGTTATTAGGT* |
| ALD_3R | **GTCTCGTGGGCTCGGAGATGTGTATAAGAGACAG***ACCTCCAAACTCCAAATCAAAAACTCCT* |
| Nextera XT v2 (i7) | CAAGCAGAAGACGGCATACGAGAT[i7]**GTCTCGTGGGCTCGG** |
| Nextera XT v2 (i5) | AATGATACGGCGACCACCGAGATCTACAC[i5]**TCGTCGGCAGCGTC** |

*Note:* Target region sequences are italicized; overhand Illumina adaptor sequences are marked in bold; i7 – Nextera Illumina 8-base Index 1; i5 – Nextera Illumina 8-base Index 2.
